# Intravital Microscopy of the Beating Murine Heart to Understand Cardiac Leukocyte Dynamics

**DOI:** 10.3389/fimmu.2020.00092

**Published:** 2020-02-04

**Authors:** Nathaniel H. Allan-Rahill, Michael R. E. Lamont, William M. Chilian, Nozomi Nishimura, David M. Small

**Affiliations:** ^1^Nancy E. and Peter C. Meinig School of Biomedical Engineering, Cornell University, Ithaca, NY, United States; ^2^Department of Integrative Medical Sciences, Northeast Ohio Medical University, Rootstown, OH, United States

**Keywords:** intravital microscopy, cardiovascular, heart, multiphoton microscopy, leukocyte

## Abstract

Cardiovascular disease is the leading cause of worldwide mortality. Intravital microscopy has provided unprecedented insight into leukocyte biology by enabling the visualization of dynamic responses within living organ systems at the cell-scale. The heart presents a uniquely dynamic microenvironment driven by periodic, synchronous electrical conduction leading to rhythmic contractions of cardiomyocytes, and phasic coronary blood flow. In addition to functions shared throughout the body, immune cells have specific functions in the heart including tissue-resident macrophage-facilitated electrical conduction and rapid monocyte infiltration upon injury. Leukocyte responses to cardiac pathologies, including myocardial infarction and heart failure, have been well-studied using standard techniques, however, certain questions related to spatiotemporal relationships remain unanswered. Intravital imaging techniques could greatly benefit our understanding of the complexities of *in vivo* leukocyte behavior within cardiac tissue, but these techniques have been challenging to apply. Different approaches have been developed including high frame rate imaging of the beating heart, explantation models, micro-endoscopy, and mechanical stabilization coupled with various acquisition schemes to overcome challenges specific to the heart. The field of cardiac science has only begun to benefit from intravital microscopy techniques. The current focused review presents an overview of leukocyte responses in the heart, recent developments in intravital microscopy for the murine heart, and a discussion of future developments and applications for cardiovascular immunology.

## Introduction

The primary function of the heart is to pump blood throughout the body via the circulatory system, delivering oxygen and nutrients to the tissues, and removing carbon dioxide and waste simultaneously. The heart is composed of four chambers, each separated by uni-directional valves, that synchronously work to cycle blood through the systemic and pulmonary circulation. The function of the heart relies on the action of contractile cells, known as cardiomyocytes, specialized conducting cells that facilitate coordinating rhythmic contraction, extracellular matrix that provide mechanical support, as well as veins, arteries, and microvasculature to supply blood to the working muscle. Importantly, the heart vascular network, known as the coronary circulation, maintains perfusion of myocardial tissue with hemodynamics that are out-of-phase to the systemic circulation (1).

Almost all diseases of the heart involve an immune response with a high degree of spatial and temporal regulation that is orchestrated by functionally varied leukocyte populations. This dynamic nature of leukocytes, coupled with the fact that contractile motion at the cell and tissue level is an essential function of the heart, present a unique and challenging environment to study the immune system. Given that the heart is highly specialized and metabolically active, containing the highest oxygen consumption rate per unit of tissue in the human body (2), it is highly susceptible to insults that decrease its function. These include myocardial infarction, a condition that is caused by the partial blockage of blood supply to the myocardium, and chronic heart failure, which is a slow and progressive pathology that weakens the pumping ability of the heart. Diseases of the heart are the most common cause of death in the United States and the majority of populations worldwide (3). Furthermore, the incidence of heart disease continues to increase at an alarming rate despite significant advancements in therapies and techniques (4). Although an inflammatory component has long been recognized as a contributing factor in these diseases, the coupling between the dynamics of the inflammatory cell populations and heart function remains unexplored.

Common techniques to study the cellular basis of cardiac disease in experimental models typically capture a static point in time (e.g., post-mortem immunohistology) or use reduced preparations (e.g., *ex vivo* perfused heart, cell dissociation or isolation and analysis such as flow cytometry). These approaches have led to enormous progress in our fundamental understanding of leukocyte biology in the heart over the past century. However, these approaches fail to capture simultaneous and interacting processes at the cell-scale. The study of leukocyte dynamics in the majority of organ systems, including brain (5–7), kidney (8–10) skin (11, 12), and many more (13–17), have greatly benefitted from intravital microscopy imaging approaches by providing invaluable insight into the fundamental behavior and function of these cells during normal and diseased states. The field of cardiovascular science has started to overcome the barriers of applying intravital microscopy to the heart, a critical step in understanding the pathophysiological basis of these devastating cardiovascular diseases.

The purpose of this review is to provide an overview of leukocyte responses in the heart, outline the advances in the application of intravital multiphoton microscopy to the rodent heart, and highlight its application to investigate specific questions about leukocyte biology within the heart.

## Immune Cell Populations of the Heart

The heart of a healthy adult mouse contains the full repertoire of leukocyte populations including mononuclear phagocytes, dendritic cells, neutrophils, T cells, and B cells (18). These leukocyte classes differ in their regional location in the steady-state heart (19), likely due to specific interactions with both cardiomyocytes (20) and non-cardiomyocyte resident cells including endothelial cells (21), smooth muscle cells, and fibroblasts (22), all of which are sources of cytokines, chemokines, and growth factors.

The predominant immune cell population in the heart during healthy conditions is the tissue resident macrophages, accounting for 5–10% of non-myocytes in the heart (23–25). Resident macrophages are found primarily near endothelial cells and within the interstitium between cardiomyocytes (26). Fate mapping studies have shown that cardiac macrophages arise from embryonic progenitors before the start of definitive hematopoiesis and then self-renew through local proliferation with minimal input from blood derived monocytes (26, 27). C-C chemokine receptor type 2 (CCR2) expression is low in the cardiac resident population of macrophages, however a small population of CCR2+ cardiac macrophages, and lymphocyte antigen 6C (Ly6C)+ macrophages exist in the myocardium and are thought to be derived from circulating precursors (27, 28). Histological studies demonstrate that resident macrophages have a spindle-like morphology and associate closely with cardiomyocytes and endothelial cells (20, 25). To maintain homeostasis, these cells survey the local microenvironment and can phagocytose dying or senescent cells (23). Mice expressing green fluorescent protein (GFP) under the control of the Cx3C chemokine receptor (Cx3Cr1) promotor are commonly used to identify monocytes and resident mononuclear phagocytes including cardiac resident macrophage populations (25, 29). In other organs such as the brain, these Cx3Cr1^+/GFP^ mice were used to discover that Cx3Cr1+ cells are actively moving their processes in the normal state and respond within minutes to injury (30–32). Tissue resident cardiac macrophages have recently been discovered to have tissue-specific functions in both health and disease that are not only essential for a coordinated response to injury, but also vital for healthy, steady-state cardiac physiology. Hulsmans et al. (20) demonstrated that resident macrophages are denser in the atrioventricular node, where they actively couple to cardiomyocytes to facilitate electrical conduction through connexin-43-containing gap junctions. Inducible transgenic ablation of these resident macrophages resulted in atrioventricular block, demonstrating a previously unknown, tissue specific function of macrophages. Intravascular patrolling monocytes that are Cx3Cr1+ were observed in the mouse heart during steady-state conditions (33), and are thought to rapidly infiltrate during inflammatory conditions, as has been described in other tissues (34, 35). Mast cells, dendritic cells, B cells, and regulatory T cells are found sparsely in normal cardiac tissue, while neutrophils and monocytes are not observed in myocardial tissue unless within the coronary circulation or in response to a stimulus (19, 27).

The heart is susceptible to a wide range of injuries, both acute and chronic in their nature, that initiate leukocyte responses that aim to repair. Acute myocardial infarction occurs due to an occluded or ruptured coronary artery causing ischemia to a region of the heart that would otherwise be perfused. This event initiates a coordinated immune response that can be divided into inflammatory and reparative phases which differ in leukocyte composition and phenotype (18). The inflammatory phase occurs shortly after ischemia and involves the degranulation of resident mast cells (21), the release of cytokines and chemokines including interleukin (IL)-1, IL-6, tumor necrosis factor-alpha (TNF-α), and CC-chemokine ligand 2 (CCL2) from resident macrophages and cardiomyocytes (36–38), hematopoietic growth factors from fibroblasts (39), and activation of endothelial cells to upregulate adhesion molecules (40, 41). Together these factors recruit neutrophils and monocytes from the circulation and hematopoietic stem and progenitor cell populations from the bone marrow (23, 42, 43). Within the infarcted heart, neutrophils and monocytes remove dead and dying cells by efferocytosis (44) and the release of proteolytic enzymes to facilitate digestion of dead tissue (45, 46). These actions further enhance inflammation by the production of cytokines including TNF-α, IL-1, and IL-6 (36, 47–49). Neutrophil numbers diminish ~3–4 days post-infarction in the mouse, whereas monocytes continue to accumulate in the infarct for several days thereafter, where they differentiate into macrophages and express Ly6C^Low^ (50). The presence of neutrophils is essential for the transition to the reparative phase since the release of neutrophil gelatinase-associate lipocalin promotes a reparative macrophage polarization (51). The reparative phase is further characterized by a decreased production of inflammatory cytokines and growth factors (52), accumulation of mast cells (53), and a transition of cardiac macrophages to a reparative phenotype that secrete transforming growth factor-beta (TGF-β) and vascular endothelial growth factor (VEGF) to promote fibrosis and angiogenesis (52, 54). Interestingly, some reports suggest mast cells do not influence levels of inflammation following infarction, however are more important for restoring cardiac contractility by regulating calcium sensitization of cardiomyocyte myofilaments (53).

Immune cell dynamics in chronic and adaptive pathologies of the heart such as heart failure, are amenable to intravital imaging approaches, since these changes can be far more subtle than the rapid and intense reaction caused by acute cardiac pathologies such as myocardial infarction. Heart failure is broadly defined as a condition in which the heart muscle is unable to pump enough blood to meet the body's nutrition and oxygen demands. If the heart muscle is too weak, the fraction of blood pumped out from the left ventricle can drop below 30–35%, a condition known as heart failure with reduced ejection fraction. On the other hand, heart failure with preserved ejection fraction (HFpEF) occurs when there is a deficiency in the relaxation and filling capacity of the heart chambers (diastolic dysfunction) while maintaining a normal ejection fraction (55, 56). HFpEF is increasing in incidence with a mortality rate equal to other cardiac pathologies (57–59) and an absence of any evidence-based therapies. The etiology and risk factors are broad and unclear, and there are limited experimental animal models that recapitulate the pathology (60), making it difficult to study leukocyte function. Tissue biopsies of human HFpEF patients show that cardiac macrophages (61) and blood monocytes (62) increase, and an animal model of diastolic dysfunction and aged mice demonstrate this increase is due to monocyte recruitment and increased hematopoiesis from bone marrow and spleen (61). In an animal model of pressure overload induced by transaortic constriction (TAC) that mimics aspects of HFpEF but eventually develop a reduced ejection fraction, Nevers et al. demonstrated T-cell recruitment, increased lymphocytes and macrophages in the myocardium, and increased endothelial cell expression of adhesion molecules VCAM1, ICAM1, and E-selectin (63). Wnt-mediated neutrophil recruitment facilitates cardiac dysfunction during heart failure, which has been demonstrated by improved cardiac function following neutrophil depletion in the TAC model (64). However, the behavior and localization of these neutrophils within the microcirculation or myocardium that are responsible for this damage are unknown. Furthermore, the TAC model has been criticized as a surrogate model of HFpEF due to the acute effects of aortic constriction. More recently, Hulsmans et al. used a mouse model of diastolic dysfunction and aged mice (18–30 months old) to demonstrate that increased myocardial macrophages result from monocyte recruitment and increased hematopoiesis (61). Understanding of the relationship between the ejection fraction, the blood delivered to the rest of the body and the local blood flow within the heart itself, is also limited in heart failure models.

A major advantage of using intravital microscopy to study cardiac disease, is the ability to visualize fast and dynamic behaviors of leukocyte sub-types that can influence tissue repair. Such interactions have been described in other tissues, including neutrophil mediated dismantling of damaged vessels and the creation of channels for regrowth in liver (65), and patrolling monocyte mediated neutrophil activation (10) and effector CD4+ T cell antigen recognition (9) in the inflamed kidney. It is possible that similar actions occur within the heart, yet this remains unknown. Specific cellular interactions that could be investigated with cardiac intravital microscopy include the neutrophil-induced promotion of reparative macrophage polarization following infarction (51), and neutrophil-dependent induction of hypertrophy (64).

## Intravital Imaging of Leukocytes in the Heart: Previous Approaches

Approaches to imaging the rodent heart at cell resolution need to consider three separate aspects—surgical access, image acquisition, and pre- or post-processing of images. Previous approaches to imaging leukocytes in the heart at cell resolution used varied strategies to address these three aspects, and are summarized in Tables 1, 2.

**Table 1:** Surgical and stabilization approaches in the heart.

	**Surgical access**	**Stabilization**	**Advantages**	**Limitations**	**References**
Heterotopic heart explantation	• Right cervical or abdominal transplantation of donor heart with circulatory integration	• Stabilization chamber	• Investigating graft and host cell interactions	• Abnormal hemodynamics, low temporal resolution	(66–68)
Micro-endoscopy	• 2–3 mm intercostal incision	• Suction probe	• Reduced motion artifact • Repeat longitudinal imaging	• Reduced lateral and axial resolution due to GRIN lens	(33)
Passive stabilization	• Left thoracotomy	• 3D-printed or machined circular probe with tissue adhesive	• High spatial and temporal resolution • Wide fields of view	• Motion artifact limits visualization of single-cycle dynamics	(69–76)^*^

* *Matsuura et al. (69) achieved passive stabilization in a rat model by retracting the anterior thoracic wall and placing a suction-assisted stabilization chamber*.

**Table 2:** Imaging and reconstruction approaches in the intravital heart.

	**Image acquisition**	**Advantages**	**Limitations**	**References**
Image gating	• Laser scanning microscopy • 1–16 fps • Z-stack • External pacing of cardiac and respiratory cycles • Prospective or retrospective gating	• High spatial and temporal resolution • Wide field of view	• External control of cardiac and respiratory cycles may induce abnormalities • Restricted to stable portions of cardiac cycle	(70, 71, 73, 77)
Free running	• Laser scanning microscopy • 15–30 fps • Single z-plane	• Capture fast dynamic events	• Motion artifact limits quantification of beat-to-beat dynamics • Restricted to single z-plane	(33, 69, 70, 72)
Cardiorespiratory reconstruction	• Laser scanning microscopy • 30 fps • Z-stack • Nearest neighbor cardio-respiratory phase-space reconstruction	• High spatial and temporal resolution • Wide field of view • Image volumes visualized across full cardiac and respiratory cycles • No external pacing	• Inability to visualize beat-to-beat dynamics	(74–76)

**Figure 1:**
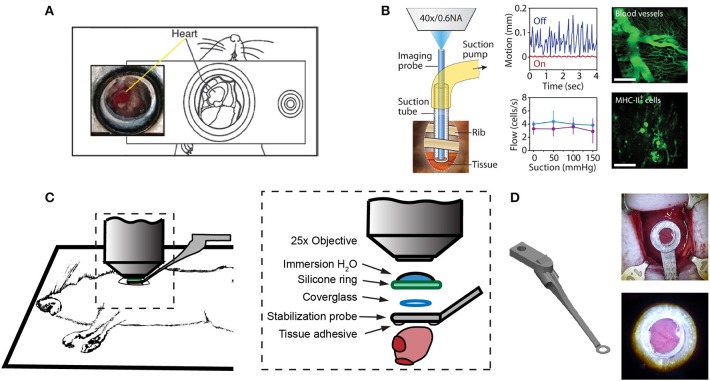
Surgical approaches for intravital microscopy of the beating mouse heart. **(A)** The cervical explanted heart model provides the benefits of studying cardiac graft vs. host interaction [image adapted from Li et al. (66) with permission]. **(B)** Endoscopic, suction-stabilized imaging provides a less invasive approach and enables time-lapse imaging that reduces motion and appears to leave major histocompatibility complex class-II (MHC-II) + immune cell numbers unchanged [image reproduced from Jung et al. (33) with permission]. **(C)** Intrathoracic approaches enable a wider field of view of the freely beating heart in mechanically ventilated animals. Optical access to the heart is gained by a left thoracotomy in the anesthetized mouse. **(D)** Passive tissue stabilization is achieved by a 3D printed stainless steel probe (left) with a coverglass and reservoir for water immersion of the microscope objective. Tissue adhesive is applied to the underside of the stabilization probe prior to attachment to the left ventricle. Photographs show the probe attached to the heart (top right) and a view of the heart surface with visible coronary vessels in the window (bottom right). Image adapted from Jones et al. (78).

One of the first approaches to imaging leukocyte populations in the mouse heart used heterotopic heart explantation with the primary application to study leukocyte recruitment into the inflamed heart (66). Heterotopic heart models are advantageous for discriminating between infiltrating leukocyte and resident cell dynamics, because the leukocyte populations of the recipient animal were labeled through transgenic techniques, whilst the donor heart remains unlabeled. Therefore, labeled leukocyte populations following transplantation are indicative of infiltrating cells and are not tissue resident cells. This technique involves transplanting a donor heart into the right cervical position of a recipient mouse, connecting the right common carotid artery to the donor ascending aorta, and right external jugular vein to the donor pulmonary artery. Optical access is gained by placing the mouse within a stabilization chamber allowing a coverglass to be lowered onto the heterotopic heart (Figure 1A). Image acquisition used video-rate scanning with 15-frame averaging, and Z-stack imaging that was partially synchronized with the heart rhythm. This method produced images of neutrophils and macrophages at baseline and following ischemia-reperfusion induced either by transplantation or coronary artery ligation. Imaging demonstrated recruitment of neutrophils to the heart, their extravasation from coronary veins, and their infiltration of the myocardium where they form large clusters (**Figure 3A**). In combination with transgenic cell ablation studies (diphtheria toxin receptor targeted expression), this technique has been used to demonstrate that tissue-resident, CCR2-expressing cardiac macrophages promote monocyte recruitment after transplantation (67). The same authors also present intravital imaging of neutrophils flowing, rolling, and crawling within coronary vessels of the murine heart in its native intrathoracic position during baseline and inflamed conditions. Intra-abdominal heart transplantation using a similar technique has also been used to image trafficking of donor dendritic cells following transplantation, finding that they migrate out of donor tissue and that this behavior is Cx3Cr1-dependent (68). While the heterotopic heart presents a unique system to study leukocyte dynamics in the context of transplant-induced ischemia-reperfusion and acute and chronic rejection, it remains restricted to these applications since the heterotopic heart does not contribute to the hemodynamics of the recipient despite perfusion of the donor coronary circulation and beating of the heart. Understanding cardiac leukocyte dynamics during healthy, steady-state conditions requires imaging of the heart within the natural intrathoracic location. More recently, a similar stabilization device enabled imaging the native heart within the intrathoracic position of rats (69). This technique involves removing the anterior chest wall and applying a circular stabilizer affixed with a cover glass and suction ring to reduce motion. Using a clever ischemia reperfusion model that was deployed during intravital imaging, the authors showed the accumulation of transplanted, GFP+, bone-marrow derived leukocytes that occlude capillaries following reperfusion.

Less invasive approaches are achievable through the use of suction-assisted, micro-endoscope, optical probes (33). A benefit of this technique is the local stabilization of the myocardial tissue that significantly reduces motion artifact and therefore eliminates the need for retrospective image processing. This technique utilizes a gradient refractive index (GRIN) lens within suction tubing, an assembly that is sufficiently small (2–3 mm) to be inserted through an incision in the intercostal space, thereby making repeat longitudinal imaging achievable (Figure 1B). To counter the small field of view typically achievable with micro-endoscopic lenses, the lens can be moved within the suction tubing via a translation stage to image multiple regions. Using repeated imaging over 6 days in Cx3Cr1^+/GFP^ mice to track monocytes and LysM^+/GFP^ mice to track neutrophils, Jung et al. provided evidence that following acute myocardial infarction, recruited monocytes come first from the vascular reservoir and then later from the spleen. Although this technique allows repeat imaging which is advantageous for studies of long-term leukocyte infiltration, the use of GRIN lenses results in a reduction in image resolution and imaging depth due to low numerical aperture (NA) and is not well-suited for studying leukocyte dynamics that involve fine cellular features. Image resolution and achievable depth rely on many factors including acquisition speed and fluorescence excitation source (multiphoton or single photon excitation), however the approximate lateral and axial resolution of GRIN lenses are 1 and 12 μm, respectively, with ≤ 0.6 NA objectives, compared to sub-micron resolution with approximately 1.0 NA objectives (79–81). GRIN lens imaging depth has been reported to be ~95 μm in brain tissue compared to ~1,000 μm without (82–85), but was not specified in heart. Typically, ~100–200 μm depth of imaging is achieved with a traditional objective in the heart (78).

Passive tissue stabilization that sufficiently reduces cardiac and respiration-induced motion in the axial direction can be used in tracking leukocyte dynamics on short-time scales with relatively wide fields of view. This has been demonstrated by Lee et al. using rhodamine-6G to label leukocytes (70). Passive tissue stabilization involves the application of a stabilizing ring that is bonded to the surface of the heart (Figures 1C,D). Image capture typically requires gating image acquisition by synchronizing with the stable portion of the cardiac cycle (diastole) (71). To eliminate motion from breathing, many studies transiently pause ventilator-induced respiration (70). Externally pacing the heart also simplifies timing image acquisition. If adequate stabilization is achieved with tissue stabilization devices so that axial motion of the cardiac contraction remains less than the axial dimension of the structure of interest (cell or capillary), then free-running images can be used to capture very short time-scale dynamics that occur over one heart beat. In our experience, cardiac contraction induced axial motion is often greater than the structure of interest, and severe suppression of motion might suggest inadequate ventricular function. Furthermore, transiently stopping ventilation of the animal, and externally pacing the heart can exert aberrant effects on the animal and heart function. Using similar passive motion stabilizers, prospective and retrospective image gating methods have been incorporated to image with high resolution (71) however, image gating fails to capture the dynamics of the heart at its peak contraction (systole). Recently, Kavanagh et al. have applied passive tissue stabilization of the beating heart without gating strategies, using fluorophore-conjugated antibodies to visualize neutrophils and platelets following ischemia-reperfusion injury (72).

## Motion Within Motion—Capturing a Moving Cell Within a Moving Organ

Motion is a defining characteristic of leukocytes, blood flow, and most importantly, the heart. The progress of cardiac leukocyte biology requires techniques to quantitatively assess leukocyte characteristics including polarized morphology, spatially-dependent speeds, and transmigration ideally with minimal artifacts and invasiveness.

Intravital microscopy of the beating mouse heart has primarily relied on multiphoton microscopy, specifically 2-photon excitation fluorescence (2PEF) microscopy, because it provides fluorescence imaging with microscopic resolution at depth in intact tissues (86). In contrast to confocal microscopy, which utilizes continues-wave (non-pulsed) laser wavelengths at which single photons can excite fluorescence, 2PEF microscopy uses photons with approximately half the energy of the confocal microscope lasers and approximately double the excitation wavelength. Therefore, it requires the nearly simultaneous (within ~10^−16^ s) interaction of two photons with a fluorescent molecule to excite fluorescence (87). The emitted fluorescence signal then scales as the square of the excitation intensity, rather than proportionally as with confocal or wide-field fluorescence microscopy, resulting in a signal primarily emitted from the beam focus where intensity is highest. An image is reconstructed by scanning this focus in the sample, measuring the amount of emitted fluorescence, and assigning that value to the image pixel corresponding to the focus position. Scattering of the emitted light does not blur the reconstructed image because all the light is known to have originated from the focus point. To achieve the higher intensities required for two-photon microscopy, the excitation laser is pulsed, and because the gaps in time between pulses are relatively long, the average power remains low. These characteristics greatly increase the signal-to-noise ratio, restrict excitation to a narrow focal volume, and minimize harmful energy deposition in the tissue. Confocal microscopy can also be used *in vivo*, but because it relies on rejection of scattered and out-of-focus light, the signal decays rapidly with depth. A further advantage of using longer wavelengths is reduced light scattering which also increases imaging depth compared to confocal microscopy.

**Figure 2:**
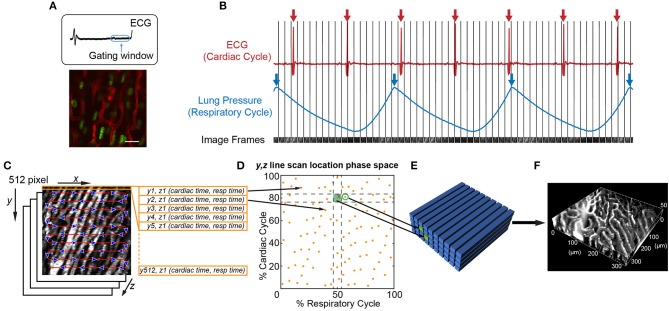
Image acquisition and reconstruction techniques for the beating mouse heart *in vivo*. **(A)** Heart structure is relatively stable during diastole allowing acquisition of full image frames with no motion artifact [image adapted from Lee et al. (70) with permission]. **(B–F)** Line-by-line reconstruction enables volumetric reconstructions at any point within the cardiac or respiratory cycles. **(B)** Simultaneous recording of the electrocardiogram (ECG) and lung pressure during imaging produces image frames at a single Z-plane that are captured at various points in the cardiac and respiratory cycles. The R-wave peak of the ECG (red arrows) and inspiratory peak of the lung pressure (blue arrows) are defined as the start and end of the cardiac cycle and respiratory cycle, respectively. **(C)** Each 512 × 512 pixel image frame is produced by raster scanning the excitation laser in the X-direction, therefore each line has a defined position in Y, a depth in Z, and a time it was captured with respect to the cardiac cycle (cardiac time) and respiratory cycle (respiratory time). **(D)** Each Y, Z line in the image is associated with a phase in the cardiac and respiratory cycles (yellow spots). An image volume is reconstructed using image lines for each Y, Z position that occur at a specified part of respiratory and cardiac cycles (green shaded box). **(E)** The closest Y, Z position (green circle) to a requested point in cardio-respiratory phase space that is absent (red cross) can be used to completely fill a three-dimensional volume. **(F)** Reconstructed vasculature of the beating mouse heart during diastole. Vasculature is fluorescently labeled with a Texas Red dye conjugated to a 70 kDa dextran. Scale bar in A represents 20 μm.

An important consideration is that leukocyte behavior varies with time, especially during inflammatory conditions. Therefore, different imaging approaches are best at capturing the intravital dynamics depending on whether the activity is faster or slower than the speed of tissue motion. For example, leukocyte crawling, extravasating, and migration through myocardial tissue typically occurs at speeds of ~7–10 μm/min (66). Since this is slower than tissue motion during systole, ~9 mm/s (5.4 × 10^5^ μm/min), and the leukocytes remain in the field of view over multiple heartbeats, imaging can be gated to limit acquisition during stable portions of the cardiac cycle (Figure 2A). However, fast leukocyte dynamics, such as intravascular flow, must account for three-dimensional movement of the tissue throughout the cardiac cycle which requires additional approaches.

It is possible to achieve capture of cell dynamics throughout the whole cardiac cycle without the need for external pacing, gating strategies, or breath holds, by using passive tissue stabilization, mechanical ventilation, and fast resonant scanning acquisition coupled with cardiorespiratory-cycle dependent image reconstruction algorithms (78). Figures 2B–F demonstrates reconstruction methods to achieve high quality volumetric images of cardiac dynamics at the microscale. Bidirectional raster scanning at a single depth relative to the microscope (Z-position) is achieved by scanning the beam focus using slow galvanometric scanning for the Y-axis, and fast resonant scanning for the X-axis, which enables the acquisition of frames at ~30 frames/s. X, Y, Z position refers to the microscope stage position and not to the orientation of the cardiac tissue since the heart is actively beating and therefore moving through the imaged region. The two primary sources of motion within acquired imaging frames are the cardiac muscle contraction and respiratory motion of the lungs pushing on the heart. Therefore, the electrocardiogram (ECG) and lung pressure are simultaneously recorded and used to index image frames according to where they occurred in the cardiac and respiratory cycles. Raster scanning of the beam focus generates a line of pixels at a specific Y-axis galvanometer position and Z-axis stage height (a single Y, Z position) at a known time relative to the cardiac and respiratory cycles. By selecting line segments that occur at a particular portion of the cardiac and respiratory cycles, image volumes can be reconstructed at any desired point during the cardiac cycle by using, for each Y, Z position in the reconstructed volume, the line scan which occurred at the requested cardiac phase. A limiting factor of this method is not all points within cardio-respiratory phase space will be sampled, which can result in missing segments within reconstructed image volumes. By using lines that are *nearest* to the desired point in cardio-respiratory phase space, complete volumetric reconstruction can be achieved. We find that ~3 s of image acquisition per imaging plane is sufficient to enable detailed volumetric reconstructions across the cardiac cycle.

**Figure 3:**
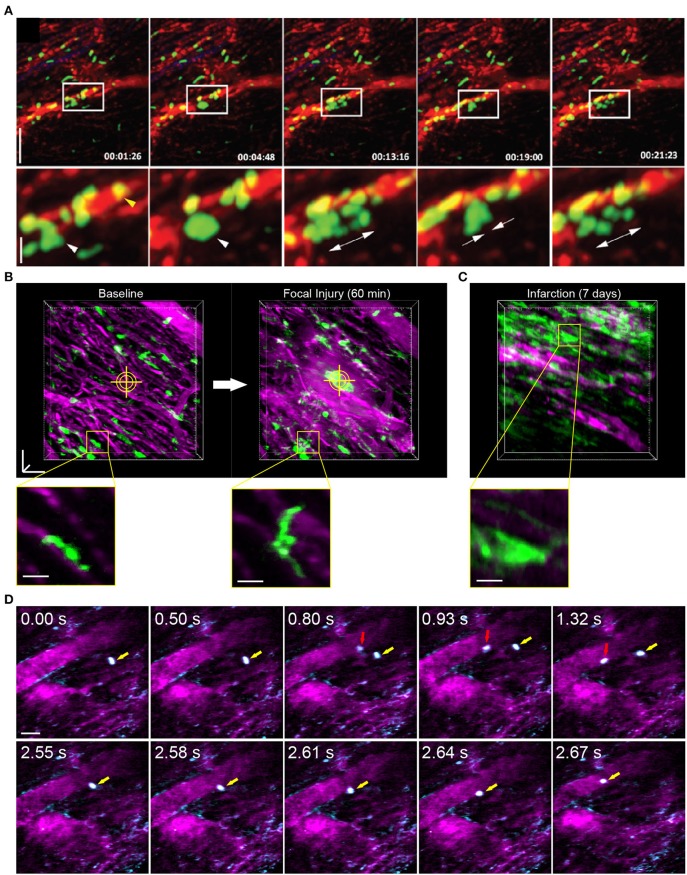
Leukocyte dynamics in the mouse heart using intravital multiphoton microscopy. **(A)** Intravital multiphoton imaging of explanted heart tissue after heterotopic cardiac transplantation into LysM^+/GFP^ mice shows neutrophil (green) infiltration from the host through vasculature labeled with non-targeted 655 nm Q-dots in red [image adapted from Li et al. (66) with permission]. Time stamp is h:min:s and scale bar is 60 μm (top) and 20 μm (bottom). **(B)** Intravital multiphoton microscopy with cardiorespiratory-dependent image reconstruction of Cx3Cr1^+/GFP^ mice hearts enables visualization of acute morphological changes of resident cardiac macrophages (green) in response to focal, laser-irradiation injury (yellow cross-hairs) over 1 h. Images are perspective Z-projections over 50 μm in depth and insets are single Z-slice of the region outlined in yellow, from 80% of the cardiac cycle and 50% of the respiratory cycle. **(C)** Intravital imaging shows increased number of macrophages in the heart following myocardial infarction compared to baseline. Vasculature is fluorescently labeled with Texas Red dextran (**B–D**, magenta). **(D)** Free-running intravital multiphoton microscopy captures intravascular rolling (red arrow) and crawling (yellow arrow) behavior of leukocytes labeled with rhodamine-6G (cyan) within vessels (magenta, Texas Red dextran). **(B–D)** Scale bars represent 50 μm, except insets in **(B)** that are 20 μm. Images captured with an Olympus XLPlan N 25 × 1.05 NA objective. All animal procedures were approved by the Institutional Animal Care and Use Committee of Cornell University.

Reconstruction methods are advantageous for tracking leukocyte dynamics that occur over slower, minute time scales, including neutrophil infiltration of explanted heart tissue [Figure 3A; (66)] and high resolution images of Cx3Cr1+ resident macrophage morphological changes in response to laser induced focal injury or following infarction (Figures 3B,C). Faster leukocyte activities including rolling and migratory behaviors can also be captured with free-running image acquisition without cardio-respiratory phase dependent reconstruction (Figure 3D). However, axial displacement due to cardiac and respiratory induced motion causes the transient disappearance of selected cells, therefore restricting the ability to capture sequential heartbeat (beat-to-beat) dynamics.

## The Future of Intravital Microscopy for Cardiac Leukocyte Dynamics

### Active Motion Compensation

Passive stabilization in combination with different acquisition and reconstruction methods, often with gating (either during imaging or at reconstruction), enables structural imaging and some measurements (70, 71, 78, 88). However, since all reconstruction methods rely on assembling image data from multiple cardiac and/or respiratory cycles, reconstructed images cannot be used to measure or visualize any process that occurs faster than the number of cycles required to generate the reconstruction, such as non-adherent leukocyte and red blood cell motion within vessels. Dynamics that change from heartbeat to heartbeat such as arrhythmic, electrical conduction irregularities in individual cells, also cannot be visualized with current reconstructions. One solution that does not involve altering the natural physiology beyond surgical access and application of an imaging window is active motion compensation, which involves moving the focal plane in synchrony with the heart motion.

The key challenge in active motion compensation is generating a feedback signal that indicates the motion of the tissue. With an appropriate feedback signal, the motion of the tissue can be matched by moving either the focal plane of the microscope, such as the microscope objective using actuators for fast motion, or moving the mouse using the microscope stage for slower motion. The feasibility of these methods was shown in early approaches using stroboscopic illumination of epicardial microvessels in combination to image, and a computer-controlled electromechanical micromanipulator that moved a micropipette in synchrony with the heart to capture phasic changes in microvascular pressure in the left ventricle of cats (89). To generate a feedback signal, image-based motion compensation methods use the alignment of successive images. In cardiac imaging, a separate camera capable of faster frame rates must be used to image a fiducial such as a fluorescent bead implanted within an imageable volume. However, this method has only been successful in correcting for in-plane motion (90) while axial movements (toward and away from the microscope objective) pose a particular challenge in cardiac imaging because such movements cause biological structures to come into and out of focus. Contact-based motion compensation methods have been proposed which use cantilever probes or strain gauges to detect three dimensional motion however, there have been no reports of successful *in vivo* imaging using laser scanning microscopy through a single cardiac cycle using this method (77). Early studies using stroboscopic illumination have measured diameter changes over 100 heat beats in small coronary vessels of the rabbit heart (91). A third motion compensation strategy takes advantage of angular changes in the reflection of a positioning laser off the surface of the tissue to measure (92) and correct for axial motion in applications such as imaging in rodent spinal cord (93). Reflective positioning offers advantages over contact-based and image-based methods as it is simple, sensitive, and does not rely on additional physical probing of the animal. The application of reflective motion compensation to cardiac imaging is a promising direction that could enable single-cycle measurements of cell trafficking and electrical activity.

### Deeper Imaging

The current maximum 2PEF imaging depth within the mouse heart is ~200 μm, limiting visualization to functions within the epicardial layer. Although this provides a good first step to studying immune cell dynamics in the heart, there are both structural and functional differences within deeper layers of the heart. Structurally, specialized conduction fibers, known as Purkinje fibers, are located in the subendocardial space, and larger coronary arteries lie deeper within the mouse myocardium. Given the recent discovery that resident macrophages facilitate electrical conduction in the heart (20), and that atherosclerosis occurs within larger coronary arteries, deeper imaging within 300 μm from the epicardial surface would enable the study of leukocyte function and behavior within these contexts.

Recent advancements in mid-infrared laser sources now make deeper imaging feasible using three-photon microscopy (94). By utilizing three excitation photons to excite a single emission photon, excitation sources with longer wavelengths can be used, and these longer wavelengths penetrate deeper into tissue and scatter much less than 2PEF wavelengths. However, the probability of three-photon interaction is low, so achieving a usable amount of fluorescence requires a higher excitation photon density or peak power. New short-pulse laser sources can now reach such peak powers at sufficient repetition rates for imaging, and provide longer wavelengths, which enable greater imaging depth (84, 94). Laser sources based on photonic crystal fibers emitting around 1,700 nm have been developed specifically for deep imaging applications (95), as well as commercially available excitation sources with turn-key optical parametric amplifiers that can be tuned from 1 to 2 μm. While able to provide more than adequate power and pulse repetition rates for three-photon imaging of slow dynamics, excitation sources in these higher wavelength ranges typically operate at a lower repetition rate (frequency of photon pulse) which presents a challenge for cardiac imaging which relies on very fast raster scanning. A resonant scanner sweeps across the focal plane so quickly that some pixels will fall between two laser pulses resulting in no signal. Current laser sources are not far off, as only 12–15 MHz is needed to guarantee at least a single pulse to acquire a 512 × 512 image at 30 fps. Continued advancements in laser technology to produce high repetition rate sources in the mid-infrared range would alleviate this problem.

Deeper imaging is also impaired by sample-dependent optical aberrations. The use of an adaptive optical element such a deformable mirror can greatly improve multiphoton microscope performance (96–98). By pre-compensating for system and sample aberrations in the excitation beam wavefront, an improved focus is achieved resulting in higher intensities and better spatial confinement. This allows for deeper penetration with greater image contrast and resolution.

### Chronic Implantable Window

Other organ systems have benefitted from chronic implantable windows to enable time-lapse imaging, making it possible to follow the same regions at the micro-scale during disease progression. This includes the cranial window (5, 6, 99, 100), dorsal skinfold chamber (101, 102), abdominal window for intestine, liver and spleen (103–105), a modified abdominal window for kidney (106, 107), and a fixation plate with micro-endoscope for femur bone marrow (108). Chronic imaging of the mouse heart would allow unprecedented visualization of pathophysiological processes in diseases of the heart, including the ability to monitor angiogenesis and fibrosis post-infarction within the full inflammatory milieu of leukocyte populations. Currently, time-lapse imaging over multiple imaging sessions for the mouse heart involves repeated surgeries for opening and closing an incision. Recently, chronic intravital imaging of the lung was achieved through a permanent window attached to a superficial portion of lung (109). This demonstrates the ability to chronically access the thoracic cavity and suggests accessing the anatomically deeper heart is feasible.

## Conclusions

With continued advancements in technology to access and image the murine heart, we are sure to make strides toward increasing our understanding of leukocyte dynamics during diseases of the heart. Targeting the immune system with therapies has proven to be successful in other diseases, especially immunotherapies for cancer (110). However, applying similar types of therapies to the varied diseases of the heart first requires an understanding of leukocyte behavior within the true *in vivo* environment which can be improved with intravital, multiphoton microscopy.

## Author Contributions

DS conceived the idea for the review, performed literature search, and wrote the manuscript. NA-R wrote the active motion compensation section. ML wrote the deeper imaging section. NN and WC provided direction for the manuscript. All authors contributed to reviewing and editing the final manuscript.

### Conflict of Interest

The authors declare that the research was conducted in the absence of any commercial or financial relationships that could be construed as a potential conflict of interest.
